# Efficacy and safety analysis of bortezomib-based triplet regimens sequential lenalidomide in newly diagnosed multiple myeloma patients

**DOI:** 10.1007/s10238-022-00879-0

**Published:** 2022-09-12

**Authors:** Qiaolin Zhou, Fang Xu, Jingjing Wen, Jing Yue, Ya Zhang, Jing Su, Yiping Liu

**Affiliations:** Hematology Department, Mianyang Central Hospital, School of Medicine, University of Electronic Science and Technology of China, No 12. Changjia Alley, Jingzhong Street, Fucheng District, Mianyang, 621000 China

**Keywords:** Multiple myeloma, New diagnosis, Sequential therapy, Bortezomib, Lenalidomide

## Abstract

The aim of this study is to analyze the efficacy and safety of sequential therapy with bortezomib-based triplet regimens without lenalidomide (PXD, including VTD, PAD, and VCD) followed by continuous lenalidomide and dexamethasone (Rd) or bortezomib and dexamethasone (Vd) treatment. The main objective is to evaluate the advantages of PXD followed by Rd compared to the combinations of bortezomib–lenalidomide–dexamethasone (VRd) in newly diagnosed multiple myeloma (NDMM). Fifty-eight nontransplant NDMM patients who were admitted to our department from 2017 to 2019 were included in this study. Bortezomib-based triplet regimens were initially selected and followed by Rd or Vd as continuous treatment once the patients achieved partial remission (PR) or better response. The efficacy and safety of the patients were observed. The Rd continuous treatment cohort was compared with historical data from the EVOLUTION trial on continuous VRd treatment. In our cohort, the overall survival rate was 100%, and progression-free survival (PFS) was 38.5% after a median of 19 (4–36) cycles of Rd continuous therapy was applied. During the follow-up period, the best outcome assessments achieved were 53.8% complete response (CR) and 84.6% excellent partial response (VGPR). A total of 23.1% had grade 3–4 or higher drug-related adverse reactions, mainly hematological toxicity, and no patients died of adverse reactions. Compared with the Vd group, the Rd group had a better PFS and VGPR rate (2-year PFS: 92.3% vs. 56.3%, *P* = 0.002; 3-year PFS: 69.2% vs. 8.0%, *P* < 0.001; VGPR: 84.6% vs. 69.2%, *P* = 0.02). No significant differences were found in ORR (100% vs. 92.3%) or CR (53.8% vs. 35.7%, *P* = 0.082). Compared with the EVOLUTION study, patients in the Rd group had a more advanced disease stage (stage III rate of 40% vs. 19%, *P* = 0.039) and worse physical status (KPS 50–60 rate of 25.0% vs. 2.0%, *P* = 0.000). However, a higher proportion of ORR (100% vs. 73.0%, *P* < 0.001), VGPR or better (75.0% vs. 32.0%, *P* < 0.001), and PFS at 12 months (90.0% vs. 68%, *P* = 0.011) were achieved. Sequential administration of bortezomib-based triplet regimens without lenalidomide as an initial therapy followed by Rd as a continuous treatment may not be inferior to VRd for first-line treatment in NDMM patients.

## Introduction

Multiple myeloma (MM) is one of the most frequent hematological malignancies. The introduction of two types of cornerstone new drugs, proteasome inhibitors (PIs) represented by bortezomib and immunomodulatory drugs (IMIDs) lenalidomide, has greatly improved the outcome of multiple myeloma patients. Based on the different and synergistic pharmacological mechanisms of lenalidomide and bortezomib, the phase 3 clinical trial SWOG S0777 confirmed that the combination of bortezomib and lenalidomide (VRd) would bring deeper responses and a higher response rate and improve the prognosis [1]. Therefore, both the NCCN and Chinese guidelines regard the VRd regimen as a preferred first-line therapy for NDMM [2, 3]. However, due to multiple factors, such as medical insurance, the economy, and society, the popularity of the VRd regimen is limited [4]. In China, the simultaneous use of bortezomib and lenalidomide was covered by the National Medical Insurance Catalog until 2019. Therefore, there are a large number of MM patients initially treated with bortezomib-based triplet regimens without lenalidomide (PXD), followed by continuous treatment with Vd or Rd [5].

It is unknown whether sequential administration of bortezomib-based triplet regimens without lenalidomide (PXD) as initial therapy and followed by Rd as continuous treatment has similar efficacy to VRd. Mateos MV et al*.* designed a phase 2 study to investigate the efficacy and feasibility of VMP (bortezomib plus melphalan and prednisone) and Rd (lenalidomide plus low-dose dexamethasone) for 18 cycles in a sequential or alternating scheme in 118 patients over 65 years of age who were not candidates for ASCT. The results showed that the probability of best complete response (CR) was 42%, and the 3-year overall survival (OS) was 72% in the sequential group [6]. Another study from Japan showed that first-line treatment with 4 cycles of VMP (bortezomib plus melphalan and prednisone) followed by continuous Rd (lenalidomide plus low-dose dexamethasone) in a phase 2 study in transplant-ineligible elderly NDMM patients resulted in an overall response rate (ORR) of 88.1% and a CR rate of 36.1%, with fewer adverse reactions of peripheral neuropathy [7]. EVOLUTION, the first registered clinical trial of VRd, resulted in an ORR of 85% and in a CR of 24% [8]. These results show that sequential or simultaneous approaches with bortezomib and lenalidomide may bring similar outcomes. However, as reported, the high discontinuation rate of 19% (8/42) due to grade 3–4 adverse effects (AE) and unsatisfactory tolerance may limit the simultaneous use of bortezomib and lenalidomide [8]. Therefore, our study aims to explore the clinical efficacy, safety, and health economic significance of bortezomib-based triplet regimens without lenalidomide (PXD) followed by continuous Rd in nontransplant NDMM patients, especially in standard-risk patients.

## Methods

### Patients

In this study, clinical data were collected from 58 nontransplant NDMM patients who were treated with a bortezomib-based three-drug regimen without lenalidomide followed by Rd or Vd as a continuous treatment in our hospital from 2017 to 2019. The diagnosis of MM was established following the criteria of the International Myeloma Working Group (IMWG) [2]. Autologous hematopoietic stem cell transplantation was ineligible or rejected by patients.

### Study treatment

**Fig. 1:**
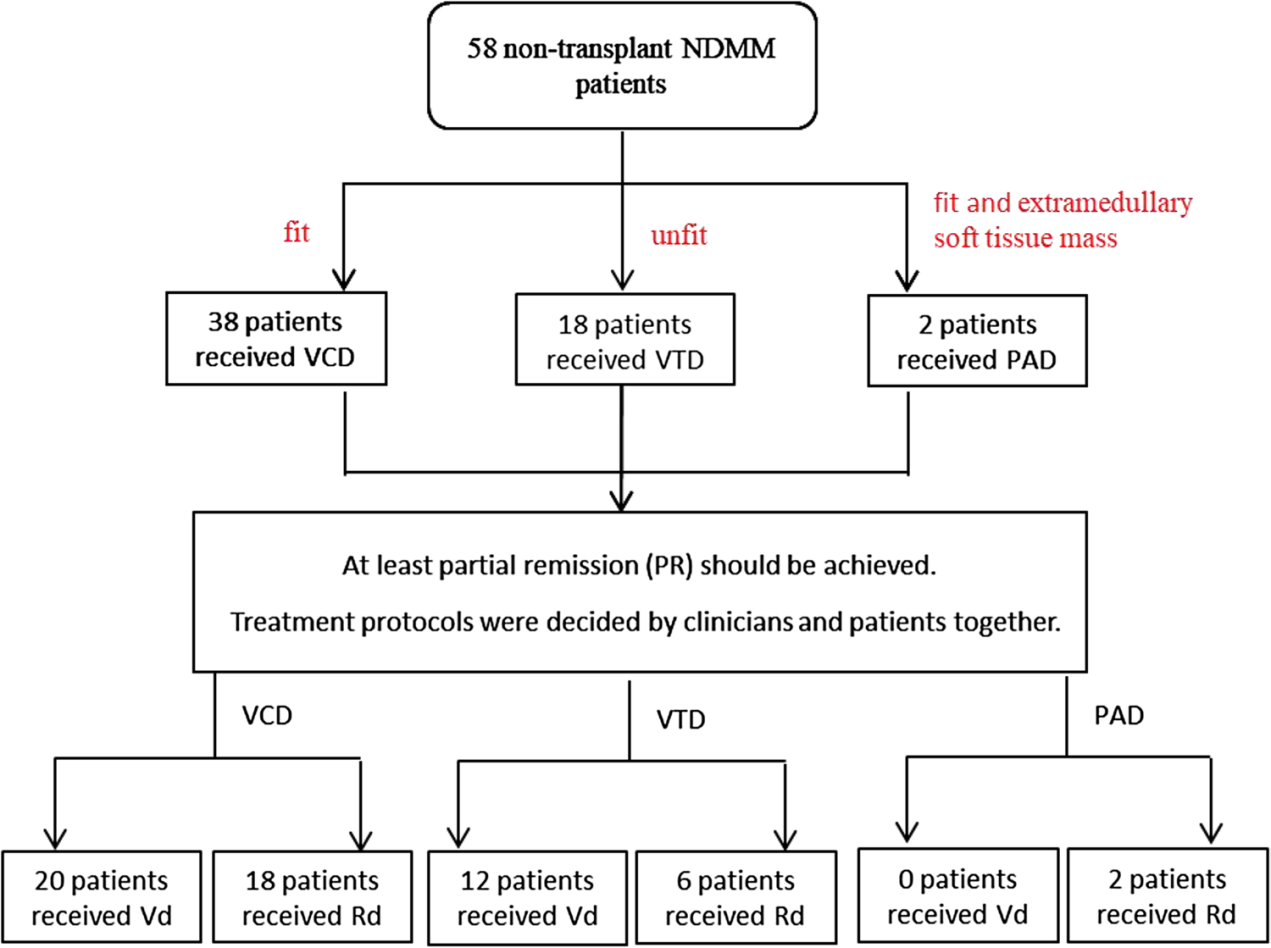
The consort diagram of reasons for the choice of the different “induction” triplets as well as the reasons for choosing Rd rather than Vd continuously

Patients were initially assigned to VCD (bortezomib plus cyclophosphamide and dexamethasone), VTD (bortezomib plus thalidomide and dexamethasone), or PAD (bortezomib plus doxorubicin and dexamethasone) that was followed by Rd or Vd as continuous treatment once they achieved partial remission (PR) or better. VCD was comprised of 35-day cycles of subcutaneous bortezomib 1.3 mg/m^2^ on Days 1, 8, 15, 22; intravenous cyclophosphamide 300 mg/m^2^ on Days 1, 8, 15; and intravenous dexamethasone 20 mg on Days 1, 2, 8, 9, 15, 16, 22, and 23. VTD was comprised of 35-day cycles of subcutaneous bortezomib 1.3 mg/m^2^ on Days 1, 8, 15, 22; intravenous dexamethasone 20 mg on Days 1, 2, 8, 9, 15, 16, 22, and 23; and oral thalidomide 100 mg daily. PAD was comprised of 35 day cycles of subcutaneous bortezomib 1.3 mg/m^2^ on Days 1, 8, 15, 22; intravenous dexamethasone 20 mg on Days 1, 2, 8, 9, 15, 16, 22, and 23; and intravenous doxorubicin 25 mg/m^2^ on Day 1. Rd was comprised of 25 mg oral lenalidomide once daily for 21 days or 10 mg once daily for 21 days plus 20 mg of oral dexamethasone once a day on Days 1, 8, 15, and 22 of each 28-day cycle. Vd was comprised of 2-week cycles of subcutaneous bortezomib 1.6 mg/m^2^ and intravenous dexamethasone 20 mg. The reasons for the choice of the different “induction” triplets as well as the reasons for choosing Rd rather than Vd continuously are shown in Fig. 1. Acyclovir (400 mg twice a day) was given to each patient treated with bortezomib to prevent herpes zoster. The patients receiving thalidomide or lenalidomide were given aspirin for thrombosis prophylaxis if there were no treatment-related contraindications. All the treatment protocols were decided by consensus between the clinicians and patients.

### Assessments

Response assessments were performed according to the IMWG response criteria [9]. The main objective of this analysis was PFS, and the other primary objectives were the best response after treatment, including ORR (partial response or better), CR, VGPR, and PR. Other outcomes were the safety of treatment, including moderate adverse reactions and severe adverse reactions, which were graded according to the National Cancer Institute Common Terminology Criteria for Adverse Events (CTCAE), version 3.0 [10].

### Follow-up

All patients were followed-up for disease progression, recurrence, or death. The last follow-up of the surviving patients was on December 6, 2021. Cases lost to follow-up were excluded.

### Statistical methods

SPSS (version 23.0) was used for all analyses. Patient-defined demographics, disease characteristics, and treatment details were analyzed using descriptive statistics. Measurement data are expressed as *χ* ± *S* or median. Categorical variables and continuous variables were analyzed using Fisher’s exact test and the Wilcoxon run-sum test, respectively. The log-rank test and Kaplan–Meier method were used to estimate the time-to-event endpoints and between-group comparisons for PFS and OS. All *P* values were two-sided; *P* < 0.05 was considered to indicate a statistically significant result.

## Results

### Patient background

Fifty-eight patients with symptomatic NDMM were included who received a bortezomib-based three-drug regimen without lenalidomide as an initial therapy. Achieving at least partial remission (PR) or better, 26 patients were treated with the Rd regimen as continuous treatment, and 32 patients were treated with the Vd regimen. Baseline demographic and disease characteristics are summarized in Table 1. The baseline characteristics were almost balanced between the Rd and Vd groups. There were more patients with stage III ISS (38.5% vs. 12.5%) in the Rd group (Table 2).

**Table 1:** Baseline clinical characteristics of the patients

Characteristic	All patients (*N* = 58)
Age (year), *n* (%)	
18–64	40 (69.0)
65–74	18 (31.0)
Median age (range)	61 (21–75)
Male, *n* (%)	28 (48.3)
ISS stage at diagnosis, *n* (%)	
I	20 (34.5)
II	24 (41.4)
III	14 (24.1)
Cytogenetic abnormalities (%)	
Not acquire	38 (65.5)
Del (17)	2 (3.4)
*t*(4; 14)/*t*(14; 16)/*t*(14; 20)	0
1q21	8 (13.7)
Other	6 (10.3)
ECOG performance status, *n* (%)	
0–2	54 (93.1)
3–4	4 (6.9)
Karnofsky Performance Status (KPS), *n* (%)	
90–100	12 (20.7)
70–80	38 (65.5)
50–60	8 (13.8)
Lactate dehydrogenase (> 190U/L), %	28 (50.0)
Extramedullary disease, %	12 (66.7)

**Table 2:** Comparison of clinical features and outcome data between Rd and Vd maintenance

Clinical features of patients	Rd maintenance	Vd maintenance	*P*
Median age (range)	53 (21–74)	63 (39–75)	
≥ 65, *n* (%)	6 (23.1)	12 (37.5)	.238
ISS stage at diagnosis, %			.022
I and II	16 (61.6)	28 (87.5)	
III	10 (38.5)	4 (12.5)	
ECOG performance status, *n* (%)			
≥ 2	6 (23.1)	2 (6.3)	.123
LDH > 190U/L, %	12 (46.2)	16 (53.3)	.969
High-risk cytogenetic abnormalities ^a^, %	2 (33.3)	6 (37.5)	.276
efficacy			
ORR (≥ PR), *n* (%)	26 (100)	24 (92.3)	
≥ VGPR	22 (84.6)	18 (69.2)	.020
CR	14 (53.8)	10 (35.7)	.082
12-month PFS, *n* (%)	26 (100)	32 (100.0)	
24-month PFS, *n* (%)	24 (92.3)	18 (56.3)	.002
36-month PFS, *n* (%)	18 (69.2)	2 (8.0)	.000
Adverse effects			
Grade 3 or above drug-related AE	6 (23.1)	8 (25.0)	.865
Grade 3 or above hematological AE	2 (7.7)	0 (0)	.197

^a^High-risk cytogenetic abnormalities were detected by fluorescence in situ hybridization (FISH) or metaphase cytogenetics, including del 17, *t*(4; 14), *t*(14; 16), and 1q21

### Treatment outcome

A total of 26 patients received Rd as a continuous treatment after a median of 6 (range 4–9) cycles of bortezomib-containing triplet induction therapy (18 cases of VCD, 6 cases of VTD, 2 cases of PAD). In the Vd group, 32 patients received a median of 8 (range 4–10) cycles of bortezomib-containing triplet induction therapy (20 cases of VCD, 12 cases of VTD, 0 cases of PAD). At the data cutoff, 53.8% (14/26) of patients remained on Rd continuous treatment, the median cycle was 19 (range 4–36), 31.3% (10/32) of patients remained on Vd continuous treatment, and the median cycle was 11 (range 4–22). In the Rd group, the reason for the remaining 46.2% of patients who discontinued treatment was disease progression. No one experienced AE discontinuation. In the Vd group, 37.5% of patients discontinued treatment for disease progression, and 6.25% discontinued treatment for severe infection.

The details of the response and comparison of efficacy between the Rd and Vd groups are shown in Table 2. The median PFS was 47 months in Rd group, the median PFS was 35 months in Vd group. The median OS was not reached in Rd group, the median OS was not reached in Vd group. At the data cutoff date, a higher proportion of 24-month PFS and 36-month PFS was observed in the Rd group than in the Vd group (92.3% vs. 56.3%, *P* = 0.002; 69.2% vs. 8.0%, *P* ˂ 0.001).

**Fig. 2:**
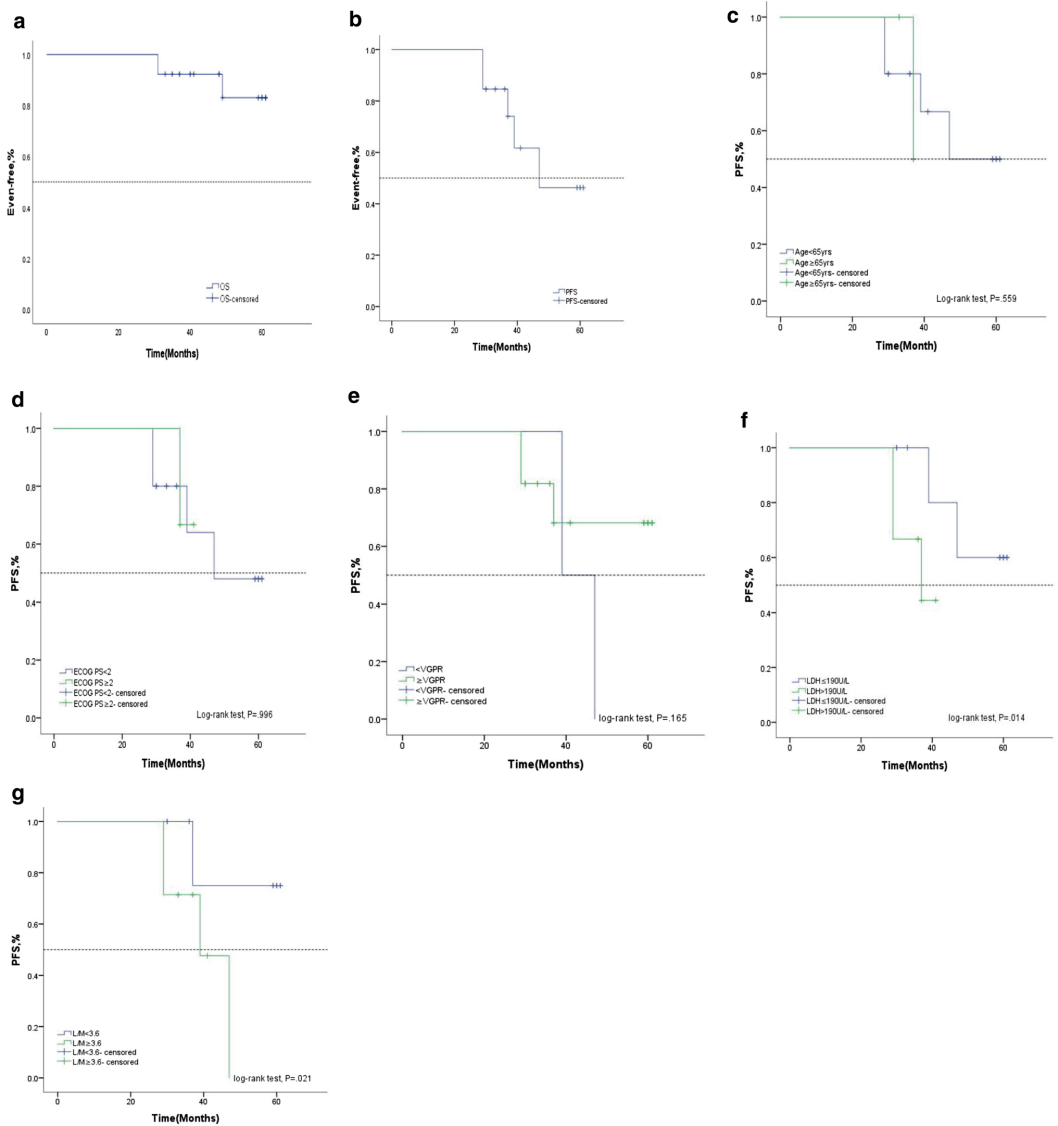
Kaplan–Meier survival curves. **a** Overall survival (OS), **b** progression-free survival (PFS); PFS according to risk stratification by age **c**, ECOG performance status **d**, VGPR **e**, LDH **f**, and LMR **g**

In the Rd group, the median follow-up was 32.8 months (5.2–71.4 months), and the OS and PFS of the entire cohort are shown in Fig. 2A and Fig. 2B. The 12-month survival rate was 100%, and the 24-month survival rate was 69.2%. No significant differences in PFS were found between patients older than 65 years and not (Fig. 2C), having ECOG PS ˃2 and less (Fig. 2D), achieving response at least VGPR and not (Fig. 2E). However, patients with higher LDH levels ˃190 U/L (Fig. 2F) and higher lymphocyte count/monocyte count ratios (LMRs) ≥ 3.6 at diagnosis (Fig. 2G) seemed to have inferior PFS (*P* ˂ 0.05) in the subgroup analysis.

### Safety

Table 2 summarizes the safety profile of those who received Vd and Rd continuous treatment. Severe adverse events (≥ Grade 3) were reported in 23.1% of patients in the Rd group, including thrombocytopenia (2 patients, 7.7%) , fitigue (2 patients, 7.7%), and constipation (2 patients, 7.7%). In the Vd group, the severe adverse events (≥ Grade 3) included infection (2 patients, 6.3%), diarrhea (2 patients, 6.3%), and constipation (4 patients, 12.5%). Peripheral neuropathy (PN) was observed in 8 patients, but there was no grade 3/4 peripheral neuropathy (PN) in the Rd and Vd groups. There were no patients who died of adverse reactions and discontinued treatment in the Rd group. Compared with Vd therapy, the Rd continuous treatment group had a similar grade 3 to 4 hematologic toxicity (*P* = 0.197) and drug-related adverse events (*P* = 0.865). Treatment was discontinued due to AEs in 2/32 patients (6.3%) during Vd continuous treatment, which was due to severe infection.

## Discussion

This retrospective study highlights the real-world efficacy, feasibility, and tolerability of sequential therapy with bortezomib-based triplet regimens without lenalidomide (PXD) followed by continuous Rd treatment in patients with NDMM. We speculate that PI-based triplet regimens as initial therapy followed by IMIDs and dexamethasone as continuous treatment may be superior to PI-based continuous therapy. Sequential administration of bortezomib and lenalidomide during different stages of treatment may bring more benefit for the patients than only one of the new agents that was used during the initial and continuous treatment.

In our study, the Rd cohort achieved a deeper treatment response and better survival outcomes than the Vd continuous treatment group. The combination of different mechanisms of PIs and immunomodulatory drugs may explain the difference. The mechanism of action of lenalidomide includes degradation of the transcription factors Ikaros (IKZF1) and Aiolos (IKZF3), which act as central transcription factors regulating various genes involved in the survival of myeloma cells. In addition, lenalidomide enhances the activation of various immune cells, including CD4, CD8, and NK T cells, while inhibiting regulatory T cells [11, 12]. While the mechanism of action of bortezomib is different, bortezomib induces apoptosis in multiple myeloma cells and triggers immunogenic cell death (ICD), characterized by the exposure of calreticulin on dying multiple myeloma cells, phagocytosis of tumor cells by dendritic cells, and induction of multiple myeloma–specific immunity [13]. Furthermore, Ganesan et al. [12] concluded from an in vitro study that when PIs and immunomodulatory drugs were combined, they exhibited synergistic activity against myeloma cells. They observed that both IKZF1 and IKZF3 were degraded at 12 and 24 h when these drugs were combined and found that there was an increase in the induction of apoptosis. Based on the different pharmaceutical mechanisms of the two agents, we supposed that during initial treatment, bortezomib could reduce the tumor burden by inducing the apoptosis of myeloma cells. As the disease is in remission, the introduction of lenalidomide may continue to induce apoptosis of tumor cells and simultaneously recover the functions of immune cells to achieve immunological surveillance. The benefits of the sequential strategy of bortezomib and lenalidomide were confirmed to be superior to only PIs as the backbone strategy in this study.

Mateos et al. [6] showed the feasibility of a sequential strategy of 9 cycles of VMP (bortezomib plus melphalan and prednisone) followed by 9 cycles of Rd in nontransplant NDMM. The outcome reported that the probability of best complete response (CR) was 42%, and the 3-year overall survival (OS) was 72%. No significant differences were found compared to their previous report in treatment response and survival [1]. Similar results were acquired in another Japanese study, which had an 88.1% overall response rate (ORR) and a 36.1% CR rate [8]. Our study further confirmed the efficacy of the sequential strategy of bortezomib and lenalidomide in NDMM despite different regimen combinations. This finding suggests that the sequential strategy could be considered an upfront treatment in NDMM patients. We also compared our study to the EVOLUTION trial, a phase 2 clinical study about VRd treatment in NDMM [8]. Although it was not a head-to-head study, the comparison gave some clues. Despite these differences in baseline characteristics, treatment response and survival outcomes in our study were comparable to those in the EVOLUTION trial, with an ORR of 100%, ≥ VGPR rate of 75%, CR rate of 45% and a 12-month PFS of 90% in our cohort compared with 73%, 32%, 7% and 68% in the EVOLUTION trial, respectively. The results suggest that the bortezomib-based triplet combination followed by continuous Rd therapy as frontline therapy could be feasible in a real-world population in NDMM. Of note, despite the promising efficacy results in our whole cohort, we still observed a relatively inferior treatment outcome in those who had high-risk CA at the situation of a low detection rate of cytogenetics and FISH analysis.

Regarding the safety profile, Grade ≥ 3 drug-related AEs were observed in 30.0% of our cohort, which was lower than the 60% reported in the EVOLUTION trial (*P* = 0.007). Of note, no Grade > 2 PN was observed in our cohort, while slightly higher rates of Grade 3 neuropathy were reported in the EVOLUTION trial. Nineteen percent of patients discontinued treatment due to AEs in the EVOLUTION study [8] and 23% in the SWOG S0777 trial [1]. This indicates that the sequential use of bortezomib and lenalidomide in induction and continuous treatment may reduce the side effects and risks of treatment discontinuation. This result was consistent with previous studies using sequential treatment [7, 8]. Subcutaneous injection (SC) and the once-weekly schedule of bortezomib in our study may be the reason for the significantly lower rates of peripheral neuropathy, which was also reported in several studies [8, 14–16], and most importantly, the efficacy of SC is not compromised compared with IV administration [17]. Additionally, Toor et al*.* [18] reported that grade III/IV peripheral neuropathy (PN) was more associated with the Vd and IMiD combination group (15.7%) than the VCd group, in which no grade III/IV PN was noted. The concurrent use of bortezomib and lenalidomide may be associated with a higher incidence and severity of peripheral neuropathy, but the pathophysiological and molecular mechanisms are unknown. Third, herpes zoster prophylaxis for patients treated with proteasome inhibitors is also one probable reason for the lower rate of severe neuropathy. Many studies have proven that antiviral prophylaxis is effective and safe in reducing the incidence of herpes zoster events [19, 20], which may reduce treatment discontinuation due to herpes zoster infection. In our study, only 6.9% suffered from herpes zoster infection, and two of the cases occurred before treatment. No patients discontinued treatment due to adverse effects in Rd group. These facts together indicated that the sequential administration of bortezomib and lenalidomide resulted in significantly lower rates of peripheral neuropathy and other side effects. In contrast to strictly controlled and well-managed clinical trials, the treatment compliance of patients may be worse in the real world. Therefore, fewer adverse reactions may be the key to decreasing treatment discontinuation and ensuring treatment efficacy.

In conclusion, for newly diagnosed MM patients, sequential administration of bortezomib-based and lenalidomide-based therapy may not be worse than the VRd regimen with simultaneous application of two drugs. A sequential strategy could lead to fewer nonhematological adverse reactions and higher patient compliance. NDMM patients may also benefit from sequential administration of bortezomib and lenalidomide due to combining the therapeutic advantages of proteasome inhibitors and immunomodulators. It is not inferior to the VRd regimen, in which bortezomib and lenalidomide were applied simultaneously. Therefore, the sequential strategy is a feasible treatment strategy for NDMM patients in the real world and can be recommended.

## Data Availability

The datasets used and/or analyzed in the current study are available from the corresponding author on reasonable request.

## Abbreviations

PXD: Bortezomib-based triplet regimens without lenalidomide
Rd: Lenalidomide and dexamethasone
Vd: Bortezomib and dexamethasone
VRd: Bortezomib–lenalidomide–dexamethasone
NDMM: Newly diagnosed multiple myeloma
PR: Partial remission
PFS: Progression-free survival
CR: Complete response
VGPR: Excellent partial response
MM: Multiple myeloma
PIs: Proteasome inhibitors
IMIDs: Immunomodulatory drugs
OS: Overall survival
VMP: Bortezomib plus melphalan and prednisone
ORR: Overall response rate
AE: Adverse effect
IMWG: International Myeloma Working Group
VCD: Bortezomib plus cyclophosphamide and dexamethasone
VTD: Bortezomib plus thalidomide and dexamethasone
PAD: Bortezomib plus doxorubicin and dexamethasone
CTCAE: Common terminology criteria for adverse events
LMR: Lymphocyte count/monocyte count
LDH: Lactate dehydrogenase
SC: Subcutaneous injection
PN: Peripheral neuropathy
